# Genetic diversity of *msp3α *and *msp1*_b5 markers of *Plasmodium vivax *in French Guiana

**DOI:** 10.1186/1475-2875-8-40

**Published:** 2009-03-11

**Authors:** Vincent Véron, Eric Legrand, Joséphine Yrinesi, Béatrice Volney, Stéphane Simon, Bernard Carme

**Affiliations:** 1 Laboratoire Hospitalo-Universitaire de Parasitologie et Mycologie Médicale, Equipe EA3593, UFR de Médecine de l'Université des Antilles et de la Guyane, Cayenne, French Guiana; 2 Institut Pasteur de la Guyane, Centre National de Référence sur la Chimiorésistance du Paludisme, Cayenne, French Guiana

## Abstract

**Background:**

Reliable molecular typing tools are required for a better understanding of the molecular epidemiology of *Plasmodium vivax*. The genes *msp3a *and *msp1*_block5 are highly polymorphic and have been used as markers in many *P. vivax *population studies. These markers were used to assess the genetic diversity of *P. vivax *strains from French Guiana (South America) and to develop a molecular typing protocol.

**Methods:**

A total of 120 blood samples from 109 patients (including 10 patients suffered from more than one malaria episode, samples were collected during each episode) with *P. vivax *infection were genotyped. All samples were analysed by *msp3a *PCR-RFLP and *msp1*_b5 gene sequencing was performed on 57 samples. Genotyping protocol applied to distinguish between new infection or relapse from heterologus hypnozoites and treatment failure or relapse from homologus hypnozoites was based on analysing first *msp3a *by PCR-RFLP and secondly, only if the genotypes of the two samples are identical, on sequencing the *msp1*_b5 gene.

**Results:**

*msp3a *alleles of three sizes were amplified by PCR: types A, B and C. Eleven different genotypes were identified among the 109 samples analysed by *msp3a *PCR-RFLP. In 13.8% of cases, a mixed genotype infection was observed. The sequence of *msp1_*b5 gene revealed 22 unique genotypes and 12.3% of cases with mixed infection. In the 57 samples analysed by both methods, 45 genotypes were found and 21% were mixed. Among ten patients with two or three malaria episodes, the protocol allowed to identify five new infections or relapses from heterologous hypnozoites and six treatment failures of relapses from homologous hypnozoites.

**Conclusion:**

The study showed a high diversity of *msp3a *and *msp1_*b5 genetic markers among *P. vivax *strains in French Guiana with a low polyclonal infection rate. These results indicated that the *P. vivax *genotyping protocol presented has a good discrimination power and can be used in clinical drug trials or epidemiological studies.

## Background

Malaria remains one of the most serious vector diseases in the world, and there are between 300 to 500 million cases of malaria annually. Although considered less dangerous than *Plasmodium falciparum*, *Plasmodium vivax *infections occasionally cause fatal cases [1-4] and have a large economic impact in endemic countries. There are estimated to be 70–80 million cases of *P. vivax *infection annually [5].

French Guiana, an overseas French territory in equatorial South America, suffers endemic malaria with areas of moderate and high transmission. The epidemiological profiles in the two main endemic foci differ. *Plasmodium falciparum *is largely predominant along the Maroni River, western border with Surinam, particularly in "Bush Negro territory". *Plasmodium vivax *is more common in the Oyapock focus, eastern border with Brazil and in the eastern slightly inland areas with a substantial increase since mild 2001 [6]. *Plasmodium malariae *is much rarer. The inland regions of French Guiana, located between the two Border Rivers, are uninhabited with the exception of two villages. In coastal regions where three quarters of the population (205,000 inhabitants according the 2008 official censuses) live, occasional bouts of malaria, mainly imported malaria, are recorded.

*Plasmodium vivax *is characterized by relapses at various time intervals associated with the presence of hypnozoites. Investigations of relapses of *P. vivax *infection report activation of heterologous hypnozoites in East Timor [7], Thailand and India [8]. There are genetic differences between the *P. vivax *populations originating from these two continents [9,10]. So far, there are few available data on hypnozoite activation during *P. vivax *relapse in patients in South America. Therefore, a better genotypic characterization of *P. vivax *at a regional scale is necessary [11]. To develop reliable epidemiological tools, appropriate genetic markers and practical molecular techniques are needed. Althought microsatellites are the most highly discriminating markers, their development is laborious and their analysis costs expensive. Merozoite Surface Proteins (MSP) have been extensively studied. These proteins of the erythrocytic stage of the Plasmodium life cycle are potential targets for vaccine and can also be used for genetic typing [12]. *Pvmsp3a *and *Pvmsp1 *are single copy genes that are highly polymorphic and currently used for *P. vivax *genotyping [13]. The MSP3*a *protein has a molecular weight of between 148 and 150 KD, and the highly polymorphic region of the gene encodes an alanine-rich central domain. Nucleotide polymorphism of *msp3a *has been studied by PCR-RFLP in different geographical regions (Iran and Papua New Guinea [14-17]) and has also been analysed by gene sequencing in Venezuela and Thailand [18,19]. *Pvmsp1*, another well-studied gene [9,12,20-26], encodes a protein of approximately 1,720 amino acids, and sequence comparisons indicate that the gene is found as two major sequence types, known as Belem and Salvador [27]. Interallelic recombination of these two sequences has been also described [22]. The *Pvmsp1 *gene is long, so many genetic studies have focused on block 5, a polymorphic region suitable for molecular typing.

The objectives of the present study were firstly to analyse the genetic polymorphism of *Pvmsp3a *and *Pvmsp1*_b5 markers in French Guiana; and secondly to propose a protocol for distinguishing between new infection or relapse from heterologus hypnozoites and treatment failure or relapse from homologus hypnozoites. The protocol was tested with blood samples from patients with multiple malaria episodes.

## Methods

### Samples

A total of 120 venous blood samples were collected in EDTA-tubes during 2005 and 2006 from 109 symptomatic malaria patients. Samples were collected as part of routine diagnosis conducted for 52 patients consulting the Health Centers at Saint Georges, Camopi and Trois Sauts living in the malaria Oyapock focus (semi-immune population) and for 57 patients consulting the Emergency Service of Cayenne (mainly non immune patients). For ten patients (R001 to R010) included in the 57, two (and for R006 three) blood samples were studied.

*msp3a *RFLP was performed on all samples, the b5 region of *msp1 *was sequenced from the patients seen in Cayenne Hospital. All samples had been sent to the malaria reference laboratory at Institut Pasteur de la Guyane by health centres (of St Georges, Camopi and Trois Sauts) and by the Cayenne Hospital, for expertise and typing as recommended by the health authorities for malaria survey.

*Plasmodium vivax *treatment was mainly chloroquine alone, as the use of primaquine was not widely authorized in 2005 – 2006, on administrative grounds.

### DNA extraction

*Plasmodium vivax *genomic DNA was extracted from 100 μL of EDTA-treated whole blood with DNeasy Blood and Tissue kits (Qiagen, Hamburg, Germany) following manufacturer's instructions. DNA was eluted in 200 μL of Tris-EDTA buffer and kept at + 4°C until use or stored at -20°C.

### Genotyping of *P. vivax *by *msp3α *PCR-RFLP

*Msp3a *polymorphism was studied using the PCR-RFLP method described by Bruce *et al *[5]. The *msp3a *gene was amplified by nested PCR in a final volume of 25 μL; 1 μL of DNA extract was used for the first PCR, and 1 μL of the first reaction product was used in the second PCR. Seven microliters of each second PCR product was digested individually with 5 units of *HhaI *enzyme (New England Biolabs Inc, UK) by incubation at 37°C for 3 hours in a final volume of 20 μL in buffer supplied with the enzyme. DNA fragments were visualized under UV illumination after electrophoresis on 1.8% agarose gel (W/V) containing 0.25 μg/mL of ethidium bromide. Polyclonal infection can be identified either by observing on electrophoresis more than one allele of the undigested PCR product (type A, B or C) or by comparing the sum of the sizes of digested fragments with the size of the uncut fragment [7].

### Genotyping of *P. vivax *by *msp1*_b5 gene sequencing

The DNA fragment encompassing the ICB5-ICB6 region of the *msp1 *gene was amplified by PCR as described by Premawansa *et al *[15]. Briefly, the amplifications were performed in 50 μL reaction buffer containing the DNA template, 1.5 mM MgCl_2_, 2 μM of each primer ICB5 (5' CCT ACT ACT TGA TGG TC) and ICB6 (5' CCT TCT GGT ACA GCT CAA TG), 200 μM of dNTP and 1.5 unit of AmpliTaq Gold polymerase (Applied Biosystem, Foster City, CA, USA). The PCR products were visualised by 1% agarose gel electrophoresis in the presence 0.25 μg/mL of ethidium bromide. Positive amplifications were purified using Qiaquick Gel Extaction Kits (Qiagen, Hamburg, Germany) and analysed by direct double strand sequencing using BigDye Terminator Kit (Applied Biosystem, Foster City, CA) with ICB5 and ICB6 primers. Sequences reactions were performed by the society Millegen (Labege, France). The amino acid sequences were aligned with the corresponding Belem and Salvador reference sequences (GenBank accesion number: M60807 and AF435603 respectively) by using the Clustal W program [28].

### Distinction between new infection or relapse from heterologous hypnozoites and treatment failure or relapse from homogous hypnozoites

Two samples corresponding to sequential of malaria from a patient are first analysed by PCR-RFLP: i) if the genotypes found are different, this identifies the second episode as a new infection or a relapse from heterologous hypnozoites, and there is no need for further analysis; ii) if the genotypes are identical, *msp1*_b5 sequence should be determined; iii) if samples are of different *msp1*_b5 type, this identifies the second episode as a new infection or a relapse from heterologous hypnozoites; iv) if same genotypes are found, treatment failure or relapse from homologous hypnozoites can be concluded with a risk of error proportional to the allele occurrence.

This protocol was assessed for 10 patients, two (or three for R006) samples were collected during successive malaria episodes at intervals of 18 days to six months.

## Results

### Diversity of the *Pvmsp3a *gene

The *Pvmsp3a *gene was amplified from total parasite DNA obtained from 109 patients. PCR products of three lengths were found: 1.9 kb (type A) corresponding to the published sequence of the Belem strain, 1.5 kb (type B) and 1.1 kb (type C). *HhaI *digestion indicated that 15 (13.8%) of the 109 blood samples were polyclonal. The frequencies of the allelic groups in mono-infected samples (n = 94) were 85.1% for type A, 9.6% for B and 5.3% for C.

In cases of polyclonal infections, genotype identification was not possible. The PCR-RFLP patterns of all three genotypes included an approximately 1.0 kb band which was slightly polymorphic. Consistent with Mueller *et al *[7], this band was not used to distinguish patterns because the size differences were not easily resolved by agarose gel electrophoresis. *HhaI *digestion revealed nine different genotypes (A1 to A9) in allelic group A, and only one genotype in each group B and group C (Table 1). Genotypes A1, A4 and A7 were the most numerous with apparition frequencies of 27.7, 12.8 and 13.8%, representing 54.3% of the monoclonal samples; the other six genotypes (A2, A3, A5, A6, A8 and A9) together made up 30.8% of the monoclonal infections.

**Table 1:** Polymorphism of *msp3α *gene analysed by PCR-RFLP among 109 patient samples.

Allele	*n *(%)
A1	26 (27.7)
A2	6 (6.4)
A3	7 (7.5)
A4	12 (12.8)
A5	7 (7.4)
A6	6 (6.4)
A7	13 (13.8)
A8	2 (2.1)
A9	1 (1.1)
B	9 (9.6)
C	5 (5.3)
Total monoclonal	94 (100)
Polyclonal	15 (13.8)

Total	109

Note: As genotypes of polyclonal samples were not clearly identified, they were not be integrated in the frequency calculation.

### Analysis of *Pvmsp1*_b5 gene sequence

The *msp1*_b5 gene was amplified from total parasite DNA (Table 2). Amplified fragment lengths were between 630 and 720 base pairs. Seven (12.3%) of the 57 samples contained two different *msp1*_b5 sequences indicating polyclonal infection. A total of 22 different sequences were found, belonging to the three major sequence types: Belem (five variants, ten samples, 15.6%) Salvador (nine variants, 21 samples, 32.9%) and a hybrid of these two sequences (eight variants, 33 samples, 51.6%) [15,19].

**Table 2:** Polymorphism of *msp1*_b5 gene sequence among 57 patients living in non-endemic aera (littoral zone)

Allele	*n *(%)	Number of Q repetition
Belem 1	2 (3.1)	23
Belem 2	3 (4.7)	22
Belem 3	1 (1.6)	18
Belem 4	1 (1.6)	17
Belem 5	3 (4.7)	15
H 1A	16 (25.0)	21
H 1B	2 (3.1)	21
H 1C	2 (3.1)	21
H 1D	1 (1.6)	18
H 2A	1 (1.6)	22
H 2B	8 (12.5)	18
H 2C	2 (3.1)	18
H 2D	1 (1.6)	18
SAL-1 A	3 (4.7)	NA
SAL-1 B	3 (4.7)	NA
SAL-1 C	2 (3.1)	NA
SAL-1 D	1 (1.6)	NA
SAL-1 E	4 (6.2)	NA
SAL-1 F	1 (1.6)	NA
SAL-1 G	1 (1.6)	NA
SAL-1 H	5 (7.8)	NA
SAL-1 I	1 (1.6)	NA

Total	64 (100)	

Note: NA: Non Applicable

**Figure 1:**
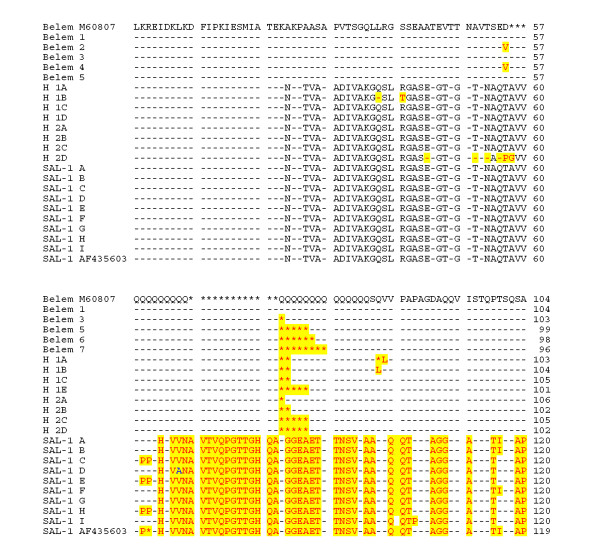
**Alignment of amino acid sequences of *Pvmsp1*_b5 gene for the 22 distinct allelic forms observed (Belem 1 to SAL-1I), part 1 of 2**. Sequences including the published Salvador type sequence (GenBank accession number AF435603) were aligned against the sequence Belem type (M60807). Drash represent identical residues and stars represent gaps.

**Figure 2:**
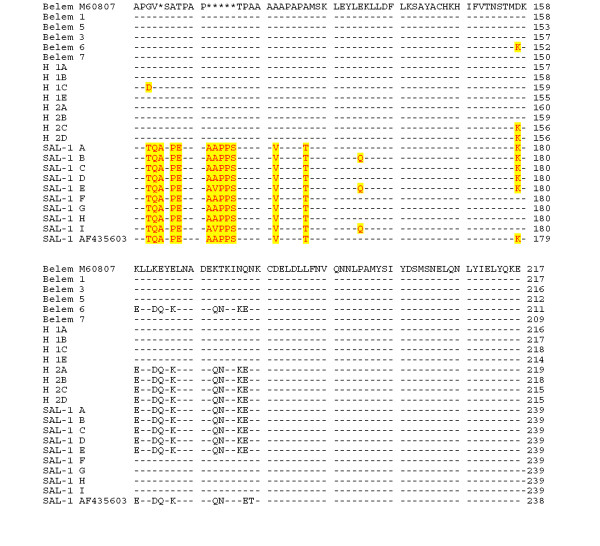
**Alignment of amino acid sequences of *Pvmsp1*_b5 gene for the 22 distinct allelic forms observed (Belem 1 to SAL-1I), part 2 of 2**. Sequences including the published Salvador type sequence (GenBank accession number AF435603) were aligned against the sequence Belem type (M60807). Drash represent identical residues and stars represent gaps.

The Belem 1 sequence was identical to the Belem M60807 sequence (see Figure 1 and Figure 2), and includes 23 Gln repetitions. The other Belem genotypes have between 15 to 23 Gln repetitions. The genotypes Belem 2 and 6 have a substitution of an aspartic acid by a valine in position 57 and the genotype Belem 4 has the same sequence as Salvador 1 between positions 151 and 171. Salvador 1 variants have similar amino acid sequences differing to Salvador reference sequence only by point mutations between positions 59 and 177. Hybrid variant sequences consist of features from both Salvador and Belem type.

Four of the 22 genotypes identified were found in 51.5% of the isolates (H1A: 25%, H2B: 12.5%, SAL-1 E: 6.2% and SAL-1 H: 7.8%) and eighteen (48.5%) with frequencies between 4.7 and 1.7%.

### Analysis of samples with the two genotyping methods

Analysis of all the data provided by both genotyping methods showed 45 different genotypes among 57 analysed samples, including 12 polyclonal infections (21%) (Table 3). Twelve of the 57 samples were A1 type; *msp1 *sequencing of these 12 samples gave four of genotype H1A, two H2B, two mixed (H1A + H2B and SAL-1 C + Belem3), one of each H1D, SAL-1 A, SAL-1 H and SAL-1 I. Among the five A4 samples, *msp3a *genotyping found four different *msp1 *genotypes, two samples being H1A. The six A7 genotype samples were all different *msp1 *alleles.

**Table 3:** Analysis with *Pvmsp3a *PCR-RFLP and *Pvmsp1*_b5 sequencing of 57 patients living in non-endemic aera (littoral zone).

Patient	*Pvmsp3a *allele	*Pvmsp1*_b5 allele
D006	A3	SAL-1 A
D149	A1	H 1D
D209	Mixed genotype	H 1A + H 2C
E012	A1	H 1A
E013	B	SAL-1 B
E015	A1	H 1A
E016	A4	Belem 2
E017	Mixed genotype	SAL-1 C + Belem 2
E021	A5	H 1A
E022	A1	H 2B
E023	A3	SAL-1 A
E029	Mixed genotype	H 2C
E030	A2	Belem 1
E037	A7	H 1A
E038	A7	Belem 4
E055	A2	H 2B
E058	Mixed genotype	H 2D
E059	Mixed genotype	H 1A + H 2B
E065	Mixed genotype	H 1A + Belem 5
E090	Mixed genotype	SAL-1 D
E096	A3	H 1A
E098	A7	SAL-1 G
E105	A5	H 1C
E119	B	H 2B
E132	A6	SAL-1 E
E134	A6	SAL-1 B
E139	A2	SAL-1 E
E141	A1	H 1A + H 2B
E142	A1	SAL-1 C + Belem 2
E153	B	H 2B
E161	A2	Belem 5
E162	B	H 2B
E170	A1	H 1A
E176	A4	H 1A
E184	A1	H 2B
E186	A4	Belem 1
F004	A1	SAL-1 A
F025	A6	H 1A
F034	A4	H 1A
F036	B	H 1B
F042	A6	H 1A
F047	A3	H 1A
F049	A7	SAL-1 E
F058	Mixed genotype	H 2A
F060	A7	H 1B + Belem 3
F118	A5	SAL-1 F
F131	A1	H 1A
R 001	A1	SAL-1 H
R 002	Mixed genotype	SAL-1 H
R 003	C	SAL-1 H
R 004	A1	SAL-1 I
R 005	C	SAL-1 H
R 006	C	SAL-1 H
R 007	A2	SAL-1 B
R 008	A4	SAL-1 E
R 009	C	H 1C
R 010	A7	Belem 5

The two genotyping methods did not identify the same polyclonal infections: there were twelve (21%) such cases, and *msp3a *genotyping identified nine (15.8%) and *msp1 *sequencing seven (12.3%); thus only four patients were identified as having polyclonal infections by both techniques.

### Genotyping of patients with multiple bouts of malaria

For 10 patients, two (or three for R006) samples were collected during successive malaria episodes at intervals of 18 days to six months (Table 4). The patients lived in the littoral zone and were contaminated during a stay along the Oyapock river. They have received a treatment with chloroquine, which has no effect on the exo-erytrocytic forms. An another stay in endemic zone, between malaria episodes, could not be excluded.

**Table 4:** Genotyping of multiple malaria episodes from ten patients.

Patient	Time between episodes	*msp3α *allele	msp1_b5 allele	Interpretation
R001		A1	SAL-1 H	
R001	18 days	C	H2C	New Infection or Relapse from Heterologous Hypnozoites
R002		C+A	SAL-1 H	
R002	6 months	C	H2C	New Infection or Relapse from Heterologous Hypnozoites
R003		C	SAL-1 H	
R003	1 month 20 days	C	SAL-1 H	Traitment failure or Relapse from homologous hypnozoites
R004		A1	SAL-1 I	
R004	2.5 months	A7	H1A	New Infection or Relapse from Heterologous Hypnozoites
R005		C	SAL-1 H	
R005	5.5 months	C	SAL-1 E	New Infection or Relapse from Heterologous Hypnozoites
R006		C	SAL-1 H	
R006	1 month 12 days	C	SAL-1 H	Traitment failure or Relapse from homologous hypnozoites
R006	5 months	C	SAL-1 B	New Infection or Relapse from Heterologous Hypnozoites
R007		A2	SAL-1 B	
R007	1.5 month	A2	SAL-1 B	Traitment failure or Relapse from homologous hypnozoites
R008		A4	SAL-1 E	
R008	1 month 5 days	A4	SAL-1 E	Traitment failure or Relapse from homologous hypnozoites
R009		C	H 1C	
R009	1.5 month	C	H 1C	Traitment failure or Relapse from homologous hypnozoites
R010		A7	Belem 5	
R010	28 days	A7	Belem 5	Traitment failure or Relapse from homologous hypnozoites

*Msp3*a genotypes of successive samples from patient R001 and R004 were different, indicating that these second episodes were due to a new infection or a relapse from heterologous hypnozoites. The first episode of the patient R002 gave a polyclonal *msp3a *genotype but the second was monoclonal; *msp1 *sequencing revealed different genotypes (SAL-1 H and H2C) in the two samples, and thus new infection or relapse from heterologous hypnozoites. For patients R003, R005, R006, R007, R008, R009, and R010, *msp3a *genotypes in the first and second samples were identical. The *msp1 *sequences from patient R005 samples were not the same (SAL-1 H and SAL-1 E), demonstrating a new infection or a relapse from heterologous hypnozoites five-and-a-half months after first epidode. Sequential samples of patients R003, R007, R008, R009 and R010 gave the same *msp1 *sequences. Genotype occurrences of samples from R007, R009 and R010 were inferior to five per cent (SAL-1 B: 4.7%, H1C: 3.1% and Belem7: 4.7%), therefore, for these patients treatment failure or relapse from homologous hypnozoites could be concluded, with a risk of error less that five per cent. This risk was 7.8 and 6.2% respectively (genotypes found were SAL-1 H and SAL-1 E) for patients R003 and R008. Patient R006 suffered three episodes of malaria: the second episode seems to have been a treatment failure or a relapse from homologous hypnozoites but with a risk of error of 7.8% (frequency of apparition of SAL-1 H, which was the genotype found in both first episodes) and the third a new infection or a relapse from heterologous hypnozoites (*msp1 *genotype found: SAL-1 B).

## Discussion

*Plasmodium vivax *diversity has been less extensively studied than that of *P. falciparum*. A better characterization of *P. vivax *populations in South America was particularly lacking. A large number of samples must be analysed to describe the genetic structure of a population, so genetic markers that are quickly and easily detectable at a reasonable cost are required. The aims of this work were to study the polymorphism of *msp3a *and *msp1*_b5 markers and to develop a method for distinguishing between new *P. vivax *infections or relapses from heterologous hypnozoites and treatment failure or relapse from homologous hypnozoites. It is of crucial importance clinically when selecting drug treatment, and in particular chosing between drugs with or without effect on the exo-erythrocytic stages. *msp3a *and *msp1 *block 5 sequence analysis indicated a high degree of polymorphism, according to different continents and countries, making these genes informative for molecular epidemiological studies. Nevertheless, they also contain conserved regions allowing amplification of the nucleotide sequences of interest. The polymorphism of *msp3a *can be evaluated rapidly at low cost by PCR-RFLP [14-17]. *Msp1*_b5 sequence reveals substantial diversity [9,12,20-27]. The RFLP technique allows a better discrimination of polyclonal infection samples than sequencing [29], so this protocol associating RFLP and sequencing could be used in regions with high rates of polyclonal infections.

Work on relapses of *P. vivax *infections demonstrates activation of heterologous hypnozoites. The genotype of the acute case is not the same as that of the recurrent infection in about 71% of relapses observed in Australian soldiers returning from East Timor [7], 61% of Thai and Burmese patients and 55% of Indian patients [8].

In French Guiana, 11 different *msp3a *genotypes were identified among 109 samples and 13.8% had mixed-species infections. Polyclonal infections were thus less frequent than described by Bruce *et al *and Cui *et al *[13,14], who reported 23% in Papua New Guinea and 19.3% of in Thailand, respectively. Three previously described types were found: in 85.1 (type A), 9.6 (type B) and 5.3% (type C) of the monoclonal samples. The frequencies of these types reported by Bruce *et al *were 70.5%, 6.7% and 22.8% and by Cui *et al *were 74.8%, 6.5% and 18.7% for A, B and C, respectively. A Venezuelan study found frequencies of 59.3 for A, 21.9 for B and 18.8% for C [18], and type C has been reported to be much more frequent in southern Iran [15] than in the studied population. Over half (54.3%) of the isolates carried one of only three alleles, A1, A4 and A7, with the other eight genotypes accounting for the remaining 45.7% of the monoclonal samples.

Polymorphism was also studied of the *msp1*_b5-6 sequence. For this protocol, genotyping *msp1 *is required in cases for which the *msp3a *genotype is identical in consecutive samples. Twenty-two different sequences in the 57 samples tested were observed. Four genotypes were strongly represented: H1A, H2B, SAL-1 E and SAL-1 H made up 51.5% of the alleles and 25% overall were H1A. The frequencies of the other 18 alleles were between 4.7 and 1.7%. A study in Iran of *msp1*_b5 sequences in 145 isolates reported 30 genotypes [26]. Seven (12.3%) mixed infections by *msp1 *sequencing, and like *msp3a *polymorphism analysis, this suggests that polyclonal infections are less frequent here than in Papua New Guinea with 38% [20]. The possible causes for this difference include malaria endemicity, population and vector density.

From these data a maximal *msp1 *allelic frequency of 5%, at which two samples from a same patient found with the same genotype can be considered to be a treatment failure or a relapse from homologous hypnozoites. In other words, relapse should be concluded with a reasonable risk of error up to 5%. If the genotype found is more represented, error risk in the conclusion of the analysis will be proportional to the genotype occurrence found.

The two methods combined revealed 45 genotypes among the 57 samples and 12 (21%) cases of polyclonal infection. Only four of these cases of polyclonal infection were identified as such by both genotyping methods and eight were identified only by one technique. Five patients were found by *msp3a *PCR-RFLP to have polyclonal infection, but *msp1 *sequencing suggested mono-infection. In cases of mixed infection with one genotype being very poorly represented in the biological sample, sequencing may not easily detect the minority genotype. In contrast, *msp3a *PCR involving nested amplification is more sensitive, and thus an additional allele should be more easily detected as it will give a 'parasite' band in addition to the known PCR band or known RFLP pattern. Thus, PCR-RFLP seems to be more sensitive than direct sequencing for detecting polyclonal infections [29]. Inversely, three patients were detected with a polyclonal infection with *msp1 *sequencing although *msp3α *PCR-RFLP detected only one genotype (two with genotypes A1 and one with A7). As the frequencies of *msp3a *genotypes A1 and A7 were high (27.7 and 13.8%, respectively), it is plausible that the two *msp1 *genotypes in these samples have the same *msp3a *genotype. The polymorphism of *Pvmsp1*_b5 and *Pvmsp3a *as described in many parts of the world, has been observed in French Guiana.

Samples from ten patients who had suffered two or three bouts of malaria (R001 to R010) were analysed. Five episodes were considered to be new infections or relapse from heterologous hypnozoites (patients R001, R002, R004, R005 and third episode for R006). Six of the second bouts were identified as being treatment failures or relapses from homologous hypnozoites. This was concluded for three patients with less than 5% risk of error and with more than 5% for also three others. The time interval between episodes for these six patients was 27 to 50 days. Molecular typing methods cannot distinguish between the both outcomes and treatment failure can occurs one month after the first episode. Therefore, based on these data, the question remains open and it is of crucial importance in clinical trials in endemic areas to know the cause of the latter episode. Moreover, to be sure that in sequential patient sample genotypes are identical, it should be verified using more efficient markers, such as microsatellites.

## Conclusion

A high diversity of *msp3a *and *msp1_*b5 genetic markers was observed among *P. vivax *strains in French Guiana and revealed a low polyclonal infection rate. The described protocol associates two molecular techniques for *P. vivax *genotyping: *msp3a *PCR-RFLP, which is a low cost method based on a highly polymorphic gene, and *msp1_*b5 sequencing, a highly discriminatory genetic tool, which provides complementary genotyping data, if required. The methodology can be easily used as a moderate- or high-throughput analysis protocol for clinical trials or epidemiological studies.

## Competing interests

The authors declare that they have no competing interests.

## Authors' contributions

VV was involved in all stages of this work and was responsible for the Pv*msp3α *PCR-RFLP and for writing the paper. EL was responsible for the supervision of the *msp1 *B5 sequencing and helped with editing the manuscript. JY, BV and SS participated in the laboratory work. BC helped with editing the manuscript. All authors read and approved the manuscript.
